# A Deep-Learning Method for Remaining Useful Life Prediction of Power Machinery via Dual-Attention Mechanism

**DOI:** 10.3390/s25020497

**Published:** 2025-01-16

**Authors:** Fan Wang, Aihua Liu, Chunyang Qu, Ruolan Xiong, Lu Chen

**Affiliations:** 1 School of Naval Architecture, Ocean and Energy Power Engineering, Wuhan University of Technology, Wuhan 430070, China; 319923@whut.edu.cn (F.W.); quchy_01@whut.edu.cn (C.Q.); 331704@whut.edu.cn (R.X.); 331651@whut.edu.cn (L.C.); 2 Sanya Science and Education Innovation Park, Wuhan University of Technology, Sanya 572024, China

**Keywords:** remaining useful life prediction, GRU, multi-feature fusion, dual-attention mechanism, power machinery

## Abstract

Remaining useful life (RUL) prediction is a cornerstone of Prognostic and Health Management (PHM) for power machinery, playing a crucial role in ensuring the reliability and safety of these critical systems. In recent years, deep learning techniques have shown great promise in RUL prediction, providing more reliable and accurate outcomes. However, existing models often struggle with comprehensive feature extraction, especially in capturing the complex behavior of power machinery, where non-linear degradation patterns arise under varying operational conditions. To tackle this limitation, we propose a multi-feature fusion model leveraging a dual-attention mechanism. Initially, convolutional neural networks (CNNs) and channel attention mechanisms are employed to preliminarily extract spatial features. Subsequently, a layer combining a Gate Recurrent Unit (GRU) and self-attention mechanisms is used to further extract and integrate temporal features. Finally, RUL values are predicted via regression. The effectiveness of the proposed method was validated on C-MAPSS datasets, and its superior performance in RUL prediction was demonstrated through comparative analysis with other methods.

## 1. Introduction

With the rapid advancement of technologies such as the Industrial Internet, the Internet of Things, and 5G, the performance and reliability of power machinery, particularly turbofan engines, have become increasingly crucial. Turbofan engines, as the core power source for modern aircraft, operate under extreme conditions and are subject to high demands for reliability to ensure safe and efficient flight. As a key component of PHM systems, the prediction of RUL plays a critical role in the operation of power machinery such as aircraft engines and their components [1,2]. The RUL prediction method is divided into a physical-based method and data-driven methods. By using existing machine learning methods and theories, data-driven methods learn information about the degradation process from the original condition-aware data and establish a mapping relationship between the degradation data and the RUL of the equipment. The data-driven method, which only relies on the learning capability of established machine learning models with little prior knowledge or detailed understanding of the equipment’s operation mechanism, has garnered widespread attention in both industry and academia, as it offers an alternative to the physical model-based methods. With the explosive growth of artificial intelligence, many machine learning algorithms have been used to predict the RUL of the equipment and have achieved notable results, such as support vector machines (SVMs) [3,4] and support vector regression (SVR) models [5].

As an end-to-end learning method, deep learning has attracted intense attention in RUL prediction. Unlike traditional machine learning methods, deep learning methods, which do not require manual feature selection, as they can automatically learn representative features from raw sensory data [6,7], have shown great promise in the RUL prediction of power machinery. Ma et al. [8] applied stacked sparse autoencoders to extract degradation features from multi-sensor monitoring data of aircraft engines, followed by logistic regression to predict the remaining useful life of the engines. Huang et al. [9] proposed two independent bidirectional LSTM models for predicting the RUL of turbofan engines. The model uses operating conditions as auxiliary input information to process degradation signals from multiple sensors. Zhai et al. [10] fused sensor data with a CNN, encoded it using a transformer, and extracted features with Long Short-Term Memory (LSTM). A contrastive learning-based tendency retention module was introduced to preserve degradation information. Validated on the NASA C-MAPSS dataset, the method showed superior prediction accuracy compared to existing methods. Zeng et al. [11] integrated attention mechanisms, Graph Attention Networks (GATs), and transformers (AGATT). The method is validated on the C-MAPSS dataset, and the results show that AGATT outperforms state-of-the-art methods in three of the four prediction tasks and achieves comparable results in the remaining one. ZHANG et al. [12] improved the traditional deep belief network and proposed a multi-target deep belief network collection method. Each depth believes that the network output occupies a certain weight on the output of the network collection. At the same time, the test demonstrates the decisive superiority of the method for analyzing NASA’s aviation engine data. Xiao et al. [13] ingeniously utilized noise to enhance the RUL prediction performance of LSTM for aircraft engines. However, two drawbacks limit the widespread application of this RUL prediction method. Kui Hu et al. [14] constructed multiple deep bidirectional recurrent neural networks (DBRNNs) with different neuron structures to extract degradation features in both forward and backward directions. These degradation features were then fed into multiple regression decision tree models for RUL prediction. DBRNN demonstrated superior performance compared to contrasting methods.

As widely studied deep learning methods, CNN and GRU are often used to capture the spatial and temporal dependencies of data in RUL prediction [15,16]. Xiao et al. [17] selected the extracted features in the time domain, frequency domain, time–frequency domain to construct the trend features, and predicted the bearing RUL by the GRU model. Shang et al. [18] directly applied convolution and pooling operations to the raw data to extract features, which were then fed into bidirectional gated recurrent units for temporal degradation information extraction. Finally, a fully connected layer was employed for RUL prediction, significantly enhancing computational efficiency. Li et al. [19] established a multi-scale CNN with a robust feature extraction ability to reflect the probability distribution of the RUL. Experimental results showed that their model performed better than other advanced models. Wang et al. [20] proposed a method combining a transformer encoder and a temporal convolutional neural network (TCNN) for RUL prediction. The transformer captures long-range dependencies, while the TCNN addresses local feature sensitivity. The model, tested on the C-MAPSS dataset, shows strong performance, particularly under complex conditions. The results demonstrated that this combined method is more accurate than using a single network for prediction. Although the aforementioned deep learning methods have achieved competitive performance, the RUL prediction method based on GRU and CNN still has some limitations: the conventional GRU only uses learned features at the last step for regression or classification. The learned features at other time steps may also have some contribution. Therefore, it is proved to be an effective method to assign weight to features reasonably. Nevertheless, the feature extraction capability of the CNN network is excellent, but it does not weigh different features, which leads to the extracted features being treated as equally important.

The self-attention mechanism (SAM) was first proposed by Bahdanau [21]. In general, the self-attention mechanism provides direct correlations between the different states of a sequence in a predictive model [22]. As the name implies, the self-attention mechanism can assign different attention weights to different parts, making the model more focused on the crucial part of the training process [23]. Fan et al. [24] proposed an end-to-end model called the Trend Attention Fully Convolutional Network to apply the attention mechanism to RUL prediction. Besides these traditional DL models, hybrid and revised DL models for RUL prediction have also been studied. Que et al. [25] connected GRU with the (Dynamic Time Distortion Warping, DTW) attention mechanism, which focuses on the weights of different time steps to improve information integrity. Yao et al. [26] introduced the attention mechanism into the GRU model and effectively predicted the remaining life of different types of rolling bearings. In conclusion, the self-attention mechanism can effectively solve the limitation of the GRU, and the combination of the GRU and self-attention mechanism can better extract features in the time dimension. Nevertheless, GRU-SAM ignores the importance of spatial dimension features. Therefore, this paper proposes an advanced RUL prediction method that leverages a dual-attention mechanism, combining CNN with the channel attention mechanism (CNN-CAM) and GRU with the self-attention mechanism (GRU-SAM). This model is designed to address the limitations of existing methods by dynamically assigning different weights to spatial and temporal features, thus improving the prediction accuracy for power machinery like turbofan engines.

Based on previous studies, this paper introduces the framework of the dual-attention mechanism. CNN-CAM can assign different weights to channels and extract features from spatial dimensions. Moreover, the GRU-SAM can further determine the importance of features and sequentially apply higher weights in the time dimension. To verify the validity of the proposed RUL prediction method, the C-MAPSS dataset was evaluated and compared with some advanced methods using the same dataset. The main contributions of this paper are summarized below.

(1) This paper proposes an RUL prediction method based on a dual-attention framework, combining CNN with GRU. The CNN-CAM assigns different weights to the channels, enhancing the focus on key features, while the GRU-SAM assigns higher weights to more crucial temporal features, thereby improving feature extraction and prediction accuracy.
(2) By combining features from both spatial and temporal dimensions, the proposed feature extraction mechanism effectively captures essential degradation information from the raw condition-monitoring data. This method fully leverages state-aware features, significantly enhancing the RUL prediction accuracy, especially for power machinery like turbofan engines.
(3) The performance of the proposed method is evaluated using the C-MAPSS dataset. The experimental results demonstrate that the proposed method significantly improves RUL prediction accuracy compared to existing advanced methods, providing an effective solution for health management and fault prediction in power machinery.

The rest of this article is organized as follows: Section 2 describes the established architecture. Section 3 discusses the experimental details and results analysis. Finally, Section 4 concludes this paper and provides some future insights.

## 2. Methodology

**Figure 1:**
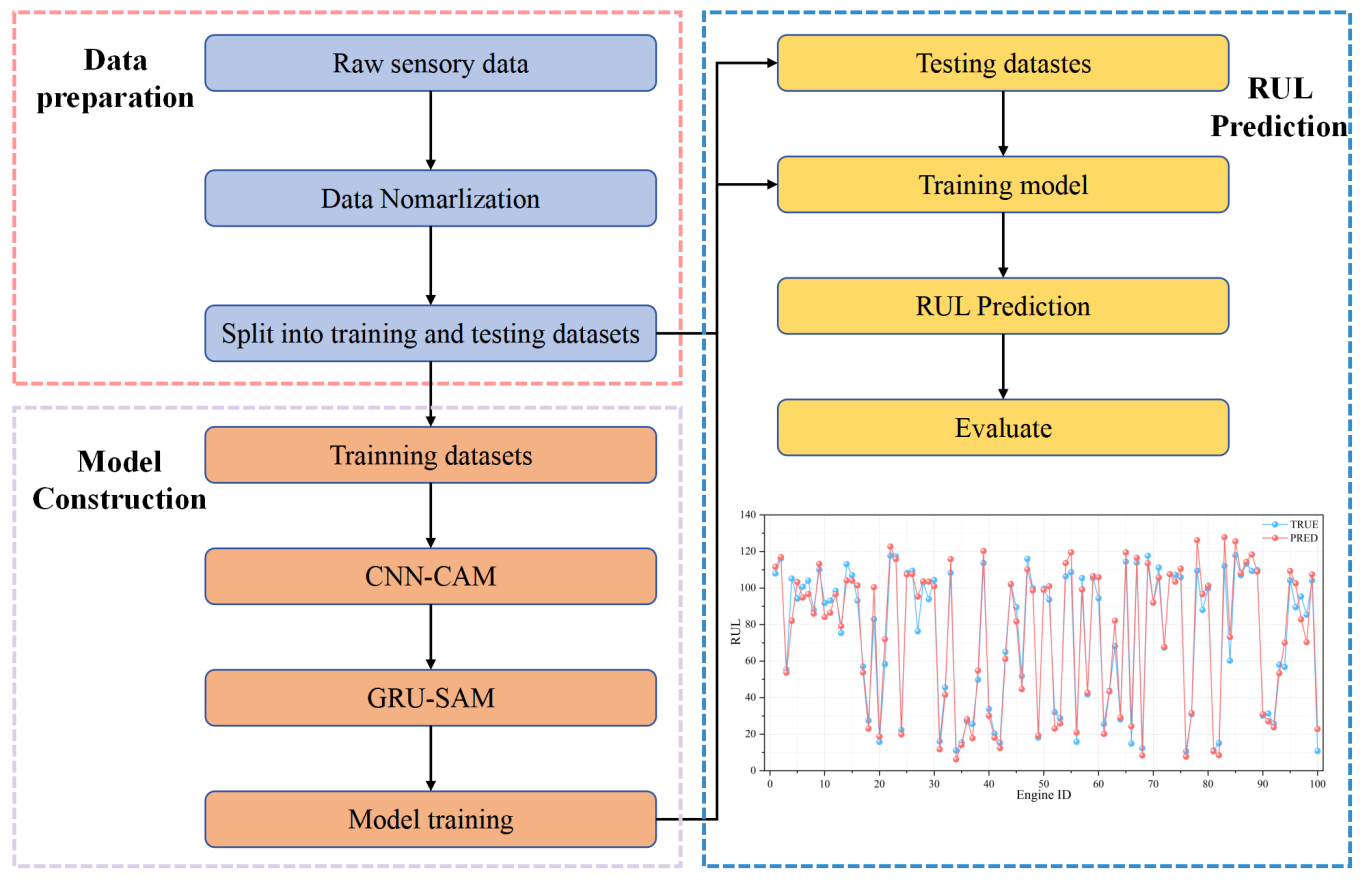
Flow chart of the proposed method.

Figure 1 shows the architecture of the proposed method, which consists of three parts: data preparation, model construction, RUL prediction. First, the raw sensory data are normalized and divided into training and datasets. Then, the training set data are input into the model for training. Finally, the model is verified, and the RUL prediction is completed with the test set.

**Figure 2:**
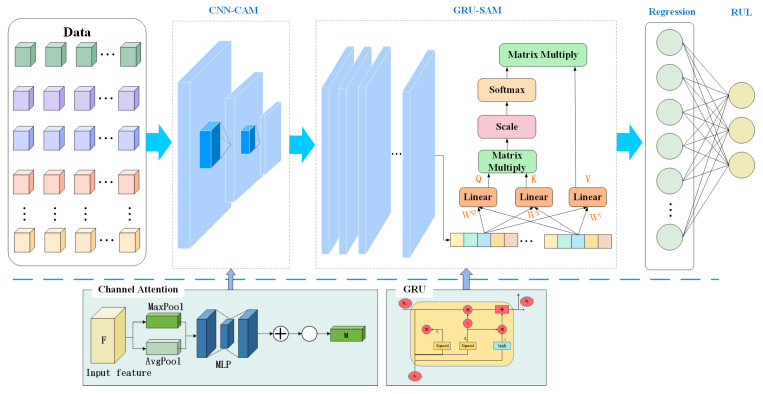
Structure of the proposed method.

In this paper, a double-attention-based model is developed to tackle the RUL prognostics. Figure 2 shows the structure of the proposed method. In this study, we propose a method comprising two key modules: CNN-CAM and GRU-SAM, each leveraging specific attention mechanisms to enhance feature extraction capabilities. Initially, the input data are processed through the CNN-CAM network. The CNN is responsible for extracting spatial features from the input data. Subsequently, these spatial features are processed through CAM, which dynamically emphasizes the most informative channels. This step ensures that the model effectively captures critical spatial features necessary for accurate predictions. Next, the features processed by the CNN-CAM module are passed to the GRU-SAM module. The GRU handles the sequential nature of the data, preserving temporal dependencies. To further optimize the extraction of temporal features, an SAM is applied. This mechanism allows the model to dynamically focus on the most relevant time steps, thereby enhancing the representation of temporal features and ensuring more accurate predictions. Finally, the fused spatiotemporal features are passed through a regression layer to achieve RUL prediction. Details of the various parts of the method are described below.

### 2.1. CNN-CAM

**Figure 3:**
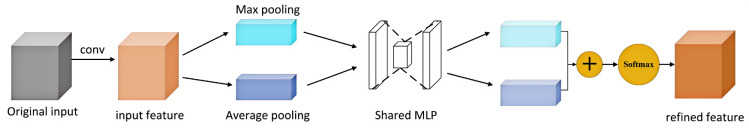
Framework structure of CNN combined with channel attention mechanism.

As a classical feedforward neural network, CNN was first proposed by LeCun [27] to solve the image processing problem. It is mainly composed of several convolution layers and pooling layers. By constructing multiple filters, the features extracted by CNN will become more and more abstract with the deepening of the network hierarchy [28]. For CNN, the original input data are convolved by multiple local filters through the convolution layer. The subsequent pooling layer extracts the essential features with fixed lengths [29]. Due to the excellent feature extraction capability of CNN-CAM, this paper proposes a framework that integrates CNN and the channel attention mechanism, as shown in Figure 3.

First, the original time series data are preprocessed to form the input data. The input data features can be expressed as follows:

(1) $$X=\left[x_{1},x_{2},x_{3},\ldots x_{k}\right],\;\mathrm{where}\;X\in R^{T\times K}$$

where $x_{k}$ denotes the $K_{\mathrm{th}}$ channel in the feature graph *X*; while *T* and *k* represent the time step and the amount of sensor data.

The convolution calculation is shown as follows:

(2) $$C_{i}=\varphi \left(W_{i}X_{i-1}+b_{i}\right)$$

where $X_{i-1}$ represents the input of the $i_{\mathrm{th}}$ convolution layer, $W_{i}$ denotes the $i_{\mathrm{th}}$ convolution kernel, and φ and $b_{i}$ represent the activation function and the function’s offset term.

Next, CAM is used to process the output data of the convolution layer. Firstly, the feature graph *X* output by the convolution layer is squeezed. Then, the spatial information in the data is integrated through the global average pooling layer and global maximum pooling layer. After extrusion, the spatial dimension of the given feature map changes from *h* × *w* × c to 1 × 1 × *c*. The generated channel descriptor corresponding to the feature graph *X* of each channel can be described as follows:

(3) $$P_{k}=S\left(X_{k}\right)=\frac{1}{h\times w}\sum_{i=1}^{h}\sum_{j=1}^{w}x_{k}\left(i,j\right)$$

where $x_{k}\left(i,j\right)$ represents the element in row *i*, column *j* of the feature graph *X*.

The channel descriptor $P_{k}$ is obtained from the feature graph after extrusion operation. The global distribution of channel feature responses is embedded in the descriptor, which helps the network utilize the information from the global receptive field at a lower level. Subsequently, $P_{k}$ is excited by the two fully connected layers and two tanh activation functions. During the excitation process, extraction descriptor *L* is generated. *L* is expressed as follows:

(4) $$L=E\left(P,FC\right)=\tanh\left(FC_{2}\tanh\left(FC_{1}P\right)\right)$$

where $FC_{1}$ is a dimension reduction layer, $FC_{2}$ is a dimension increase layer, and tanh is an activation function. Two fully connected layers reduce the model complexity through dimensionality reduction, significantly simplifying the calculation process and minimizing the number of parameters. And the tanh function sets the output weight between [−1, 1], which avoids the problem of center symmetry. Then, weighted processing is used to re-calibrate the original feature in CAM.

Finally, in order to integrate the data on the two branches of the global average pooling layer and global maximum pooling layer, the element summation is assigned to compose the output feature graph. Then, the weights are normalized by using the softmax function. Then, a scaling operation is used to assign normalized weights to the features of each channel. The final output of this module is transformed directly into the next network layer.

This paper uses CNN-CAM to extract the feature values in the spatial dimension. In order to extract the feature information more comprehensively, it is necessary to extract the feature values in the time dimension of the data. Therefore, GRU-SAM is implemented to perform feature extraction in the time dimension. The specific process is described below.

### 2.2. GRU-SAM

**Figure 4:**
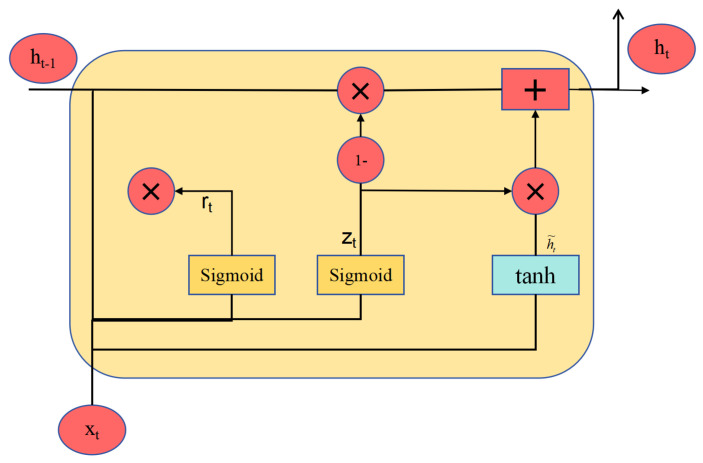
GRU network cell structure.

GRU is a type of neural network derived from recurrent neural networks (RNNs), first proposed by Cho et al. [30] Compared to traditional CNNs, GRU not only extracts temporal sequence information but also effectively addresses the vanishing gradient and exploding gradient problems inherent in conventional RNNs. The core idea of GRU is the introduction of gating mechanisms, as illustrated in Figure 4. It comprises two gating units: the update gate and the reset gate. These gating units control the flow of information within the network, allowing the selective forgetting or updating of certain information. This mechanism enables GRUs to better handle long-term dependencies while reducing the number of parameters that need to be trained. The computational formulas for the GRU model are as follows:

(5) $$z_{t}=\sigma \left(W_{z}\left[h_{t-1},x_{t}\right]\right)$$

(6) $$r_{t}=\sigma \left(W_{r}\left[h_{t-1},x_{t}\right]\right)$$

(7) $${\tilde{h}}_{t}=\tanh\left(W_{h}\left[r_{t}⊙h_{t-1},x_{t}\right]\right)$$

(8) $$h_{t}=\left(1-z_{t}\right)⊙h_{t-1}+z_{t}⊙{\tilde{h}}_{t}$$

where $z_{t}$ is the output vector at time step *t* of the update gate; $W_{z}$ denotes its weight parameters; $h_{t-1}$ represents the hidden state of the previous moment; $x_{t}$ is the input of the time step *t*; σ indicates the sigmoid activation function; $r_{t}$ is the output vector at time step *t* of the reset gate; $W_{r}$ denotes its weight parameters; $h_{t}$ is the hidden vector at time step *t*; $W_{h}$ denotes its weight parameters; tanh is the hyperbolic tangent activation function; and $h_{t}$ signifies the memorized state vector.

While the GRU network is capable of processing time series data, it can only output a fixed-length sequence and does not differentiate between features in terms of their importance. Simultaneously, for excessively long sequences, the capacity of the units to store information may need to be increased, which may result in reduced prediction accuracy. In order to solve these two problems, this paper proposes a method combining the self-attention mechanism and GRU.

Originating from the differences in the attention of the human visual system [31,32], the attention mechanism is used to allocate different feature information resources [33]. As technology evolves, self-attentive mechanisms are combined with deep learning methods and have achieved notable success. In the prediction process, the attention model will autonomously find the features that are more important to the prediction result and assign higher weights to them so as to improve the accuracy of the prediction.

In this paper, the learning feature output by the GRU network is expressed as $H={\left\{h_{1},h_{2},\ldots h_{n}\right\}}^{T}$, where *T* represents the transpose operation, and $h_{i}={\left\{x_{1},x_{2},\ldots x_{n}\right\}}^{T}$ is the self-attention mechanism’s input, and represents the data’s time step. The specific formula is as follows:

(9) $$u_{i}=\mathrm{softmax}\left(W^{T}h_{i}+b\right)$$

(10) $$a_{i}=\frac{\exp\left(u_{i}\right)}{\sum_{i=1}^{n}\exp\left(u_{i}\right)}$$

where $W^{T}$ and *b* are the weight matrix and bias terms in the attention network, $a_{i}$ is the influence of the $i_{\mathrm{th}}$ eigenvalue on the target sequence data, and all are added to 1. Then, the feature sequence is weighted to obtain the output sequence, which can be expressed as follows:

(11) $$AH=\sum_{i=1}^{n}a_{i}u_{i}$$

The integration of GRU with SAM allows the model to dynamically focus on more relevant features, enhancing the feature representation and significantly improving the model’s performance.

## 3. Experimental Study

In this section, we discuss the validation of the RUL prediction method presented in this paper and compare it with some advanced methods using generic datasets. Section 3.1 describes the details of this dataset; Section 3.2 introduces the related technologies and details of data preprocessing in detail; and Section 3.3 proposes two indexes to evaluate RUL prediction. In Section 3.4, the practical steps and determining relevant hyperparameters are elaborated. Section 3.5 discusses the effects of some hyperparameters on the results and uses ablation experiments to determine the validity of the methods studied in this paper. Finally, the results obtained in the experiments are compared with those obtained by some current advanced methods.

### 3.1. Dataset Description

**Figure 5:**
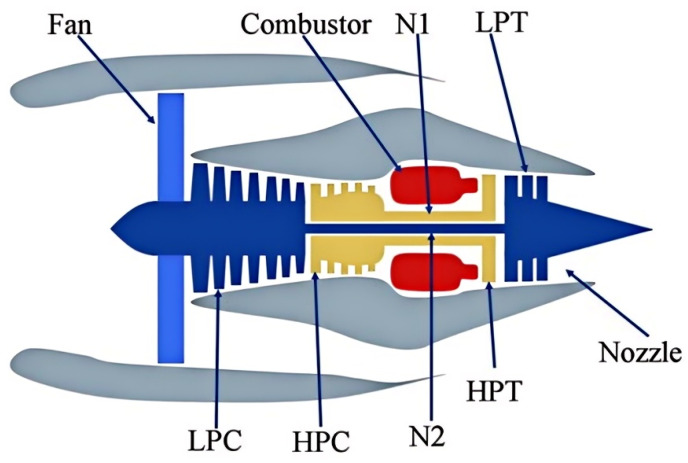
Simplified diagram of the turbofan engine.

To investigate the degradation modeling and life prediction of aero-engines, NASA used the Commercial Modular Aerospace Propulsion Simulation System (C-MAPSS) to simulate a series of performance degradation processes of major engine components (as shown in Figure 5) and made the corresponding engine performance degradation dataset publicly available [34].

The components of generate power include fans, gas chambers, low-pressure turbines, high-pressure compressors, and nozzles. The engine is designed to perform simulated operations at a sea level of 40,000 feet with 90,000 pounds of thrust, Mach numbers of 0 to 0.90, and temperatures of −60 to 103 °F. The C-MAPSS dataset includes four sub-datasets, namely FD001, FD002, FD003, and FD004. Each sub-dataset is further divided into training and test datasets. The training and test datasets are composed of the sensor data of a specific engine during each run cycle, helping to predict the true RUL of each engine in the test file with the given sensor measurements. The datasets are arranged in the *N* × 26 matrix. Each data sample includes 26 variables:

1. Engine unit number of each engine;
2. Degradation cycle of each turbofan engine;
3. Operation setting parameters of three turbofan engines;
4. Sampling data of 21 sensors on the turbofan engine in each operation cycle.

*N* is the number of signals recorded for each engine. Table 1 shows the specific information of the dataset [35].

The C-MAPSS dataset has detailed multi-sensor measurements, different operating conditions, and benchmark states in the RUL prediction field. Due to its comprehensive representation of turbofan engine degradation and widespread use in academic research, the C-MAPSS dataset was chosen in this paper to validate the proposed method.

### 3.2. Data Preprocessing

#### 3.2.1. Data Normalization

**Figure 6:**
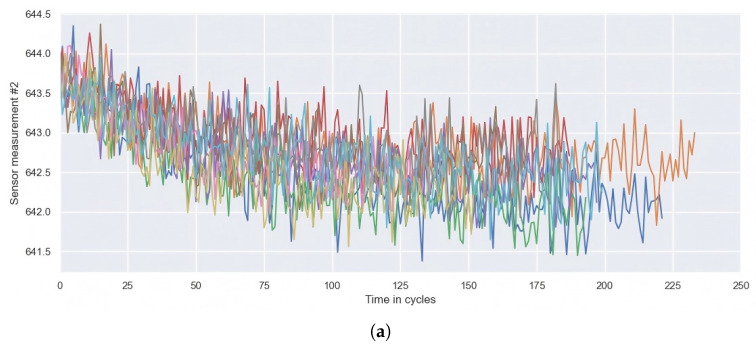

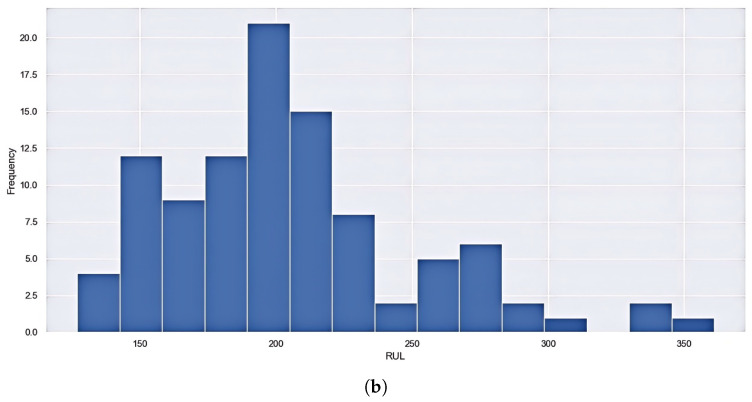
(**a**) # 2 Sensor measurement for 100 engines. (**b**) Life distribution of 100 engines.

Collected from multiple sensors, the dataset has different value ranges. Figure 6a shows the degradation data of “Sensor 2” for 100 engines in the FD001 dataset, which illustrates much noise. Figure 6b shows the lifetime distribution of 100 engines in the FD001 dataset. Since the uneven life distribution increases the computational complexity of the model, it is necessary to normalize the data to speed up the convergence and improve the prediction accuracy. In this paper, the Min–Max method is adopted to normalize the data of the training set and the test set. The formula is as follows:

(12) $$x_{i}^{\prime }=\frac{x_{i}-x_{i}^{\min}}{x_{i}^{\max}-x_{i}^{\min}}$$

where $x_{i}$ represents the value of the current time point; $x_{i}^{\max}$ and $x_{i}^{\min}$ refer to the maximum and minimum values of the current sensor at all time points, respectively; and $x_{i}^{\prime }$ denotes the data obtained after normalized calculation. The normalized data will be within the range of [0, 1].

It is essential to mention here that the sensors labeled as 1, 5, 6, 10, 16, 18, and 19 show relatively smooth behavior in the degradation experiment, which means that these data do not show regression characteristics, i.e., they do not provide significant deterioration information. Therefore, the data from these seven sensors should be excluded from this paper, and the data from the other 14 sensors will be applied to train the model proposed in this paper.

#### 3.2.2. Sliding Window Processing

**Figure 7:**
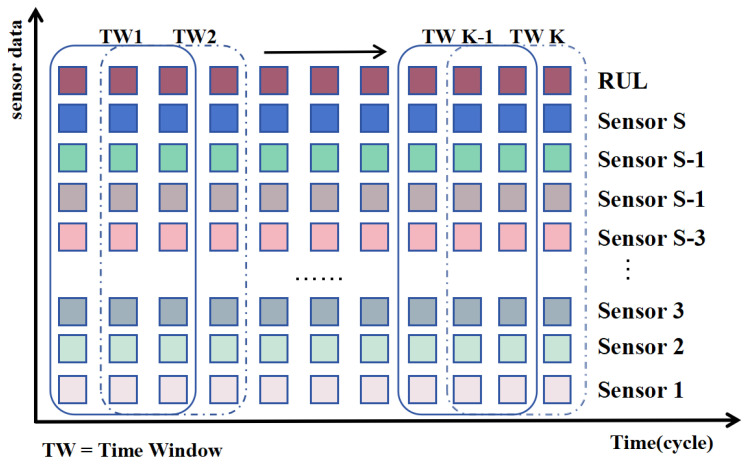
Sliding window processing.

The sliding window method is commonly employed to partition multivariate time series data, enabling the model to capture temporal dependencies across various time steps. An illustrative example of sliding window processing is depicted in Figure 7. Selecting an appropriate window length is crucial, as a window that is too large can increase the complexity of the model and reduce its practical utility, while a window that is too small may fail to adequately capture the underlying relationships in the time series. The normalized raw data, after being processed by a sliding window, become $X=\left\{x_{1},x_{2},\ldots x_{k}\right\}$.

#### 3.2.3. RUL Label Settings

**Figure 8:**
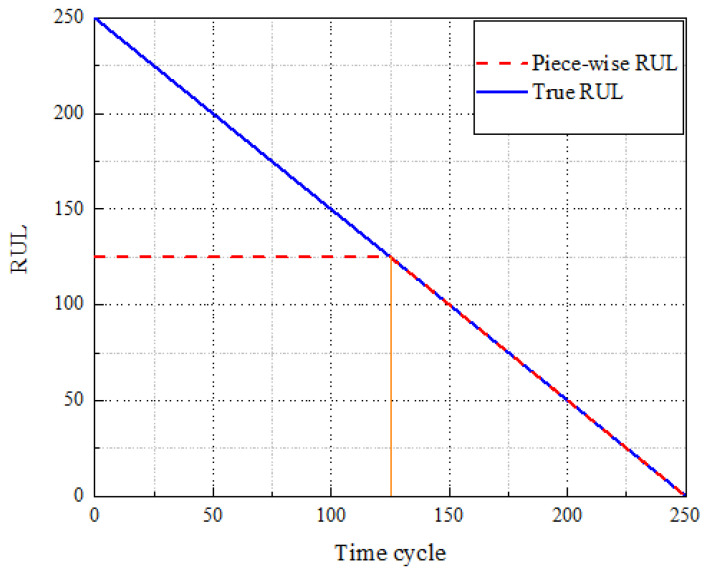
Piecewise linear model.

**Figure 9:**
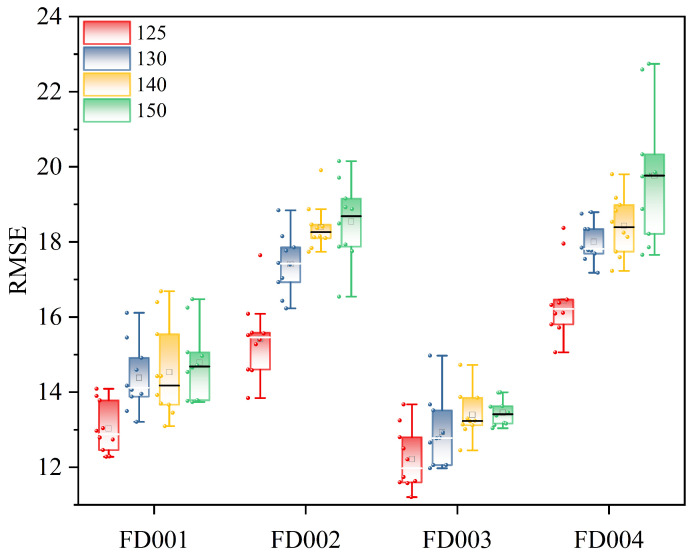
The proposed method analyzes the experimental results in different RUL values.

In the C-MAPSS dataset, sensor output data from the turbofan engine remain relatively stable during the initial phase due to the absence of faults or degradation. Hence, early prediction of remaining useful life (RUL) is not only futile but also computationally inefficient. Prior studies [36,37] indicate that a piecewise linear model can confine the maximum RUL within a specific range, thereby preventing excessive RUL predictions. Consequently, this study employs a piecewise linear model to process engine RUL, as illustrated in Figure 8. The maximum RUL is set to 125; any value exceeding 125 is uniformly capped at 125. To validate this maximum RUL setting, comparative analyses were conducted using four different values: 150, 140, 130, and 125, across four datasets. The results, depicted in Figure 9, demonstrate that a maximum RUL value of 125 yields the most accurate predictions.

### 3.3. Evaluation Metrics

In order to verify the validity and accuracy of the method, two evaluation metrics were utilized: root means square error (RMSE [38]) and score function (Score [39]). RMSE can measure the extent to which the predicted RUL value deviates from the real RUL value. At the same time, Score is an index proposed by PHM08 data competition to evaluate the predicted performance. The smaller the value of the two indexes is, the better the prediction performance will be. These two evaluation indexes will be used comprehensively to evaluate the model’s prediction performance. The formula of RMSE is as follows:

(13) $$\mathrm{RMSE}=\sqrt{\frac{1}{N}\sum_{i=1}^{N}{\left({\hat{y}}_{i}-y_{i}\right)}^{2}}$$

where *N* represents the total number of samples, and ${\hat{y}}_{i}$ and $y_{i}$ are predicted from the RUL value and the actual RUL, respectively.

The scoring function is one of NASA’s evaluation metrics for this open question. In the RUL forecast, if the predicted value is less than the actual value, it is considered an early forecast, and the subsequent decision will be more conservative based on the result. The situation where the predicted value is greater than the true value is called a late forecast, which may lead to an accident or incident that cannot be reflected by a single RMSE.

In order to improve the evaluation system, this paper introduces the scoring function, which expressed as follows:

(14) $$\mathrm{Score}=\left\{\begin{array}{c} \sum_{i=1}^{N}\left(e^{\frac{{\hat{y}}_{i}-y_{i}}{13}}-1\right),\;{\hat{y}}_{i}<y_{i}\\ \sum_{i=1}^{N}\left(e^{\frac{y_{i}-y_{i}}{10}}-1\right),\;{\hat{y}}_{i}\geq y_{i} \end{array}\right.$$

where *N* represents the total number of samples, and ${\hat{y}}_{i}$ and $y_{i}$ represent the predicted RUL value and the actual RUL values, respectively.

**Figure 10:**
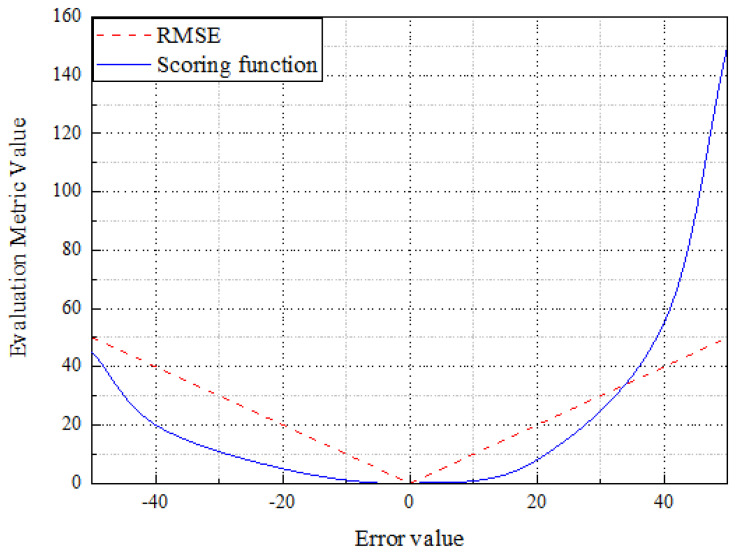
Piecewise linear model.

The relationship between Score and RMSE is shown in Figure 9. With the increase in the error between the predicted and actual values, the RMSE increases linearly, and the Score curve increases exponentially. Early prediction (left side of Figure 10) is better than late prediction (right side of Figure 10) because the goal is to prevent engine failure. The late prediction will be dominated by a single outlier, thus hiding the actual overall accuracy of the model. Therefore, in practice, RMSE and Score should be integrated to evaluate the model’s performance comprehensively.

### 3.4. Experimental Setup

To validate the accuracy and effectiveness of the model, the training data of FD001 to FD004 were tested first in this paper. The original perception data were extracted by one-dimensional convolution and put into the channel attention mechanism for adaptive weight allocation. The features output from CNN-CAM were entered as input into the network combined with GRU-SAM for training and weight allocation again. The experiment used the training set to train the model and then the test set to verify the model. The algorithm iterated over each dataset ten times (32 epochs were included in each iteration) and recorded the best RMSE and Score once in each iteration. Applying a dropout rate of 0.2 (i.e., 20% of randomly selected hidden layer neurons were ignored) helped to minimize the overfitting that tends to occur during training [40]. Finally, the best data for the iteration were saved. The hyperparameters of the proposed method are shown in Table 2.

### 3.5. Result Analysis

#### 3.5.1. Impact of Window Size

**Figure 11:**
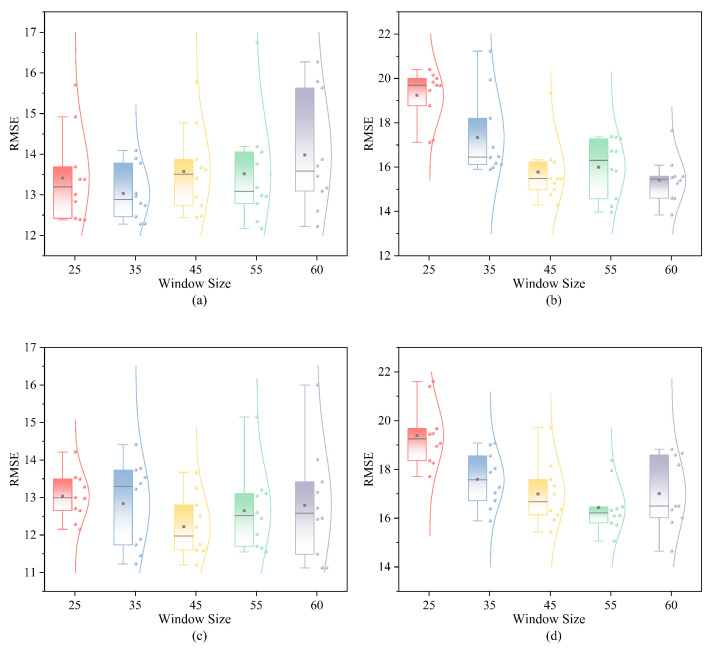
The proposed method has experimental results of different window sizes on four datasets. (**a**) FD001: RMSE. (**b**) FD002: RMSE (**c**) FD003: RMSE. (**d**) FD004: RMSE.

Window size is one of the most critical parameters in the model, directly influencing its performance outcomes. The impact of window size on the proposed performance prediction model was investigated using the FD001-FD004 datasets. Five window sizes—25, 35, 45, 55, and 60—were evaluated, with results shown in Figure 11. Overall, RMSE varied with window size, and trends differed across datasets. In the FD001 dataset, performance improved as window size increased from 25 to 35, likely due to more informative data being included. However, further increases in window size resulted in decreased predictive performance, possibly due to overfitting. As shown in Table 1, while the FD003 dataset is broadly similar to FD001, it has two operational modes, necessitating a larger window size. Therefore, more complex data may require additional information to enhance RUL prediction accuracy. This is corroborated by the FD002 and FD004 datasets, where model performance improved with increasing window size. Hence, different datasets require tailored window sizes to optimize RUL prediction accuracy. The optimal window sizes for the four datasets are summarized in Table 3.

#### 3.5.2. Ablation Study of the Proposed Architecture

**Figure 12:**
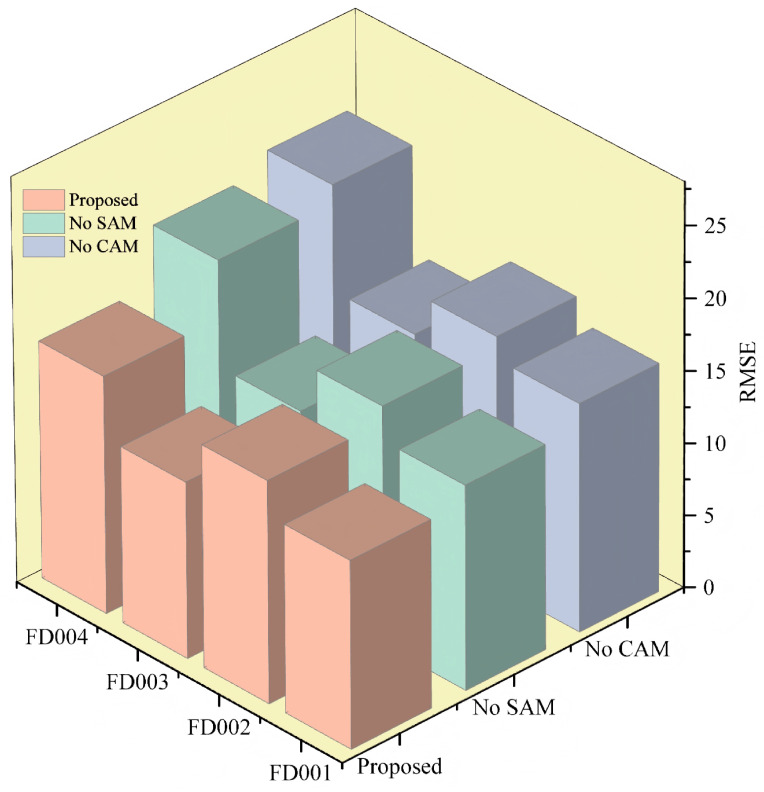
Experimental results of the ablation study.

To determine the validity of the method proposed in this paper, ablation experiments were performed for validation. This paper used the model with (or without) CAM to explore the capability of CAM and the model with (or without) SAM to analyze the role of SAM in the feature weight assignment. Specifically, three methods, including No CAM, No SAM, and the model used in this paper, were selected for the comparative study, and the experimental results are shown in Table 4 and Figure 12.

Figure 12 presents a comparative analysis of the predictive performance of various model configurations through ablation experiments. The introduction of the SAM and the CAM significantly enhanced prediction performance. This improvement is more intuitively illustrated in Table 4, where it is shown that the removal of CAM resulted in a marked decline in model performance, underscoring its crucial role in capturing key features. Although CAM had a greater impact on overall error reduction, the SAM also significantly improved model performance by capturing long-term dependencies and dynamically weighting inputs. In summary, both SAM and CAM are pivotal in enhancing predictive model performance. This study proposes that integrating these two mechanisms enables the model to perform well across all datasets, thereby validating the effectiveness and necessity of the dual-attention mechanism.

#### 3.5.3. Prognostic Results Analysis

**Figure 13:**
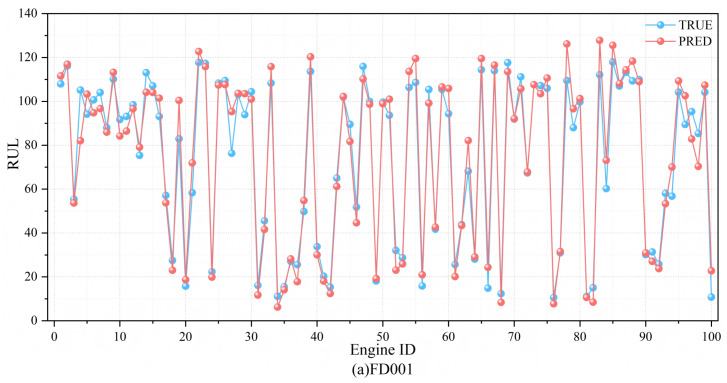

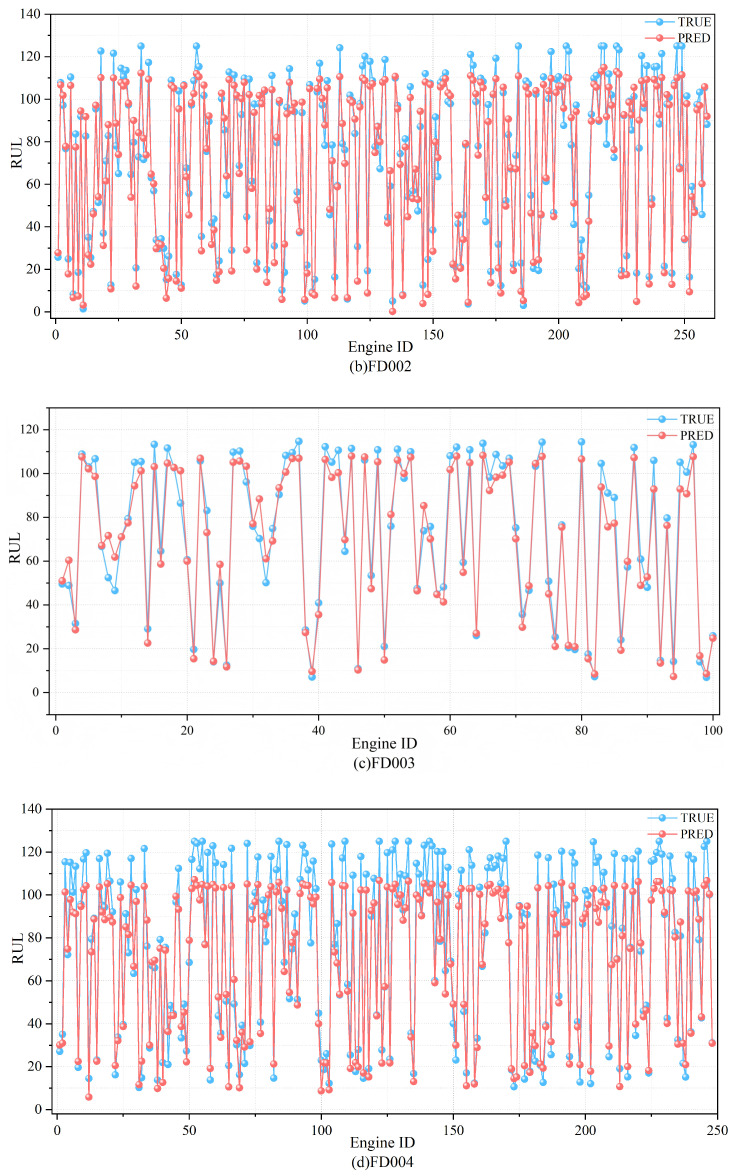
The prediction results of the method in this paper on four datasets and the real RUL fitting graph. (**a**) FD001: (**b**) FD002 (**c**) FD003 (**d**) FD004.

The predicted results and the actual RUL values are shown in Figure 13. As can be seen from the figure, the predicted RULs of the four datasets closely match the actual RUL values. The fitted curves on FD001 and FD003 are close to the actual values, showing that the model can effectively predict the RUL. On the other hand, most of the predicted results in the FD002 and FD004 datasets are smaller than the actual results, which indicates that the model is capable of significantly mitigating the risks associated with late predictions.

#### 3.5.4. Comparison with the State-of-the-Art Methods

Table 5 and Table 6 present the experimental results of the proposed method compared to several state-of-the-art methods across four datasets. Results highlighted in bold indicate the best performance among all methods.

As shown in Table 5, the proposed method achieved the lowest RMSE on three of the four datasets (FD002, FD003, and FD004), demonstrating its superior prediction accuracy. On the FD001 dataset, although the RMSE of the proposed method was slightly higher than other advanced methods such as the double-attention-based architecture [47], the overall average RMSE across all datasets (14.47) was the best among the compared methods. This indicates that the proposed method performs well in both simple and complex scenarios. In particular, the proposed method showed a significant improvement in the complex datasets FD002 and FD004, where the reduction in RMSE compared to other methods, such as MODBNE [12], was particularly pronounced. This highlights the proposed method’s robustness and adaptability in handling complex operational conditions.

Furthermore, as seen in the Score metrics of Table 6, the proposed method consistently achieved better scores across three datasets (FD002, FD003, and FD004). While the Score on FD001 was slightly worse than BLSTM-CNN [48], the proposed method still maintained a balance between prediction accuracy and early failure detection. Early predictions can help reduce maintenance costs and avoid potential damages in practical scenarios, as demonstrated by the superior performance in FD004.

In summary, compared with advanced methods like the double-attention-based architecture [47] and BiGRU-TSAM [46], the proposed method surpassed them in RMSE and Score on average, showing its ability to achieve accurate and reliable RUL predictions while maintaining a practical advantage in handling complex conditions.

## 4. Conclusions

This study proposes a feature fusion framework for RUL prediction, integrating CNN and GRU with a dual-attention mechanism. Initially, spatial features were extracted using CNN-CAM, while temporal features were subsequently extracted using GRU-SAM, enabling the fusion of spatio-temporal features to enhance feature extraction capabilities. Finally, the trained neural network model was applied for RUL prediction. In this study, we utilized the C-MAPSS datasets and evaluated our proposed method using RMSE and score function metrics, comparing it with other state-of-the-art methods. Additionally, ablation experiments were conducted to validate the necessity and effectiveness of our approach. Experimental results demonstrate that our proposed method outperformed other advanced methods, effectively integrating information on power machinery characteristics through the dual-attention mechanism. Future research will focus on two key aspects: first, exploring more advanced feature fusion techniques and incorporating additional domain knowledge to further enhance model performance; second, optimizing computational efficiency by exploring lightweight model architectures or hardware-accelerated solutions, enabling the proposed method to achieve faster training and inference times while maintaining accuracy.

## Figures and Tables

**Table 1:** Description of the C-MAPSS dataset.

Datasets	FD001	FD002	FD003	FD004
Engine units for training	100	260	100	249
Engine units for testing	100	259	100	248
Sensor measurements	21	21	21	21
Maximum cycle	362	378	525	543
Minimum cycle	128	128	145	128
Operation conditions	1	6	1	6
Fault conditions	1	1	2	2

**Table 2:** Hyperparameters of the proposed method.

Hyperparameters	Value
Number of convolution kernels for Conv	4
Activation function in CNN	Hyperbolic tangent (tanh)
GRU number of hidden units in the network	50
Number of neurons in FC layer behind GRU	[50, 10]
Window sizes for FD001 to FD004	35/60/45/60
Learning rate	0.001
Dropout	0.2
Kernel sizes for Conv1 to Conv5	1 × 3
Activation function in GRU network	ReLU

**Table 3:** Window size information of the four datasets.

Datasets	FD001	FD002	FD003	FD004
Time window size	35	60	55	60
No. of training time windows	18,231	48,819	21,820	57,763
No. of testing time windows	10,696	29,070	13,696	37,742

**Table 4:** Results of RMSE in the ablation study on four datasets.

Methods	FD001	FD002	FD003	FD004
CNN + GRU-SAM(No CAM)	15.81	17.37	14.48	21.79
CNN-CAM + GRU (No SAM)	14.18	16.54	13.12	20.51
CNN-CAM + GRU-SAM (proposed)	**13.03**	**15.41**	**12.21**	**16.43**

*Note:* **Bold numbers** represent the best performance of the list.

**Table 5:** Results of the proposed method and other methods on RMSE.

RMSE					
Methods	FD001	FD002	FD003	FD004	Average
CNN [41]	18.45	30.29	19.82	29.16	24.43
MODBNE [12]	15.04	25.05	12.51	28.66	20.315
VAE+LSTM [42]	15.88	25.78	14.29	23.93	19.97
GA+RBM+LSTM [43]	12.56	22.73	12.1	22.66	17.5125
Multi-attention+TCN [44]	13.25	19.57	13.43	21.69	16.985
ABGRU [45]	12.83	17.97	13.23	21.55	16.4
BiGRU-TSAM [46]	12.56	18.94	12.45	20.47	16.105
Doubleattention-based architecture [47]	12.25	17.08	13.39	19.86	15.645
BLSTM-CNN [48]	**12.13**	16.01	**11.96**	18.1	14.55
Proposed	13.03	**15.41**	12.21	**16.43**	**14.47**

*Note:* **Bold numbers** represent the best performance of the list.

**Table 6:** Results of the proposed method and other methods on Score.

Score				
Methods	FD001	FD002	FD003	FD004
CNN [41]	1286	13570	1596	7886
MODBNE [12]	334	5585	421	6557
VAE+LSTM [42]	322	4990	309	4720
GA+RBM+LSTM [43]	231	3366	251	2840
Multi-attention+TCN [44]	235	1655	239	2415
ABGRU [45]	221	2072	279	3625
BiGRU-TSAM [46]	213	2264	232	3610
Doubleattention-basedarchitecture [47]	198	1575	290	1741
BLSTM-CNN [48]	**174**	1230	242	1513
Proposed	217	**796**	**189**	**1029**

*Note:* **Bold numbers** represent the best performance of the list.

## Data Availability

The original contributions presented in this study are included in the article. Further inquiries can be directed to the corresponding author.
